# M6A regulator-mediated immune infiltration and methylation modification in hepatocellular carcinoma microenvironment and immunotherapy

**DOI:** 10.3389/fphar.2022.1052177

**Published:** 2022-11-10

**Authors:** Kun Zhao, Bing Wei, Yingxuan Zhang, Wenkai Shi, Guokun Zhang, Zhengfeng Wang

**Affiliations:** ^1^ Department of Hepatobiliary and Pancreatic Surgery, The First Affiliated Hospital of Zhengzhou University, Zhengzhou, Henan, China; ^2^ Department of Surgery, The First Affiliated Hospital of Zhengzhou University, Zhengzhou, Henan, China

**Keywords:** m6A regulators, hepatocellular carcinoma, tumor microenvironment, immunotherapy, prognosis

## Abstract

**Introduction:** Tremendous evidence indicates that N6-methyladenosine (m6A) epigenetic modification and m6A-related enzymes constitute a complex network, which jointly regulates prevailing pathological processes and various signaling pathways in humankind. Currently, the role of the m6A-mediated molecular regulatory network in hepatocellular carcinoma (HCC) remains elusive.

**Methods:** We recruited expression and pathological files of 368 HCC patients from The Cancer Genome Atlas cohort. Four public datasets serve as external authentication sets for nearest template prediction (NTP) validation. The correlation between 35 regulators and their prognostic value was compared. Gene set variation analysis (GSVA) was used to explore the latent mechanism. Four independent algorithms (ssGSEA, xCell, MCP-counter, and TIMER) were used to calculate the ratio of tumor cells and non-tumor cells to evaluate the tumor immune microenvironment. The m6Ascore model was established by principal component analysis (PCA). Prediction of immunotherapy and potential drugs was performed using TIDE and SubMap.

**Results:** A total of 35 m6A regulators were widely associated, most of which were risk factors for HCC patients. The m6A phenotypic-cluster revealed differences in regulator transcriptional level, gene mutation frequency, functional pathways, and immune cell infiltration abundance under distinct m6A patterns. As expected, the m6A gene cluster confirmed the aforementioned results. The m6Ascore model further found that patients in the high-m6Ascore group were associated with lower tumor purity, higher enrichment of immune and stromal cells, upregulation of metabolic pathways, lower expression of m6A regulators, and favorable outcomes. Low-m6Ascore patients were associated with adverse outcomes. Notably, low-m6Ascore patients might be more sensitive to anti-PD-L1 therapy.

**Conclusion:** This study found that a classification model based on the m6A manner could predict HCC prognosis and response to immunotherapy for HCC patients, which might improve prognosis and contribute to clinical individualized decision-making.

## Introduction

The global number of individuals dying with liver cancer and/or the corresponding complications has been increasing gradually (Chen et al., 2021c). Also, as the most widespread among primary liver cancers, hepatocellular carcinoma (HCC) is a serious threat to public safety (Pinter et al., 2021). Surgical resection is a feasible method for early HCC, but the current treatment of advanced HCC is still not optimistic (Xu et al., 2020). With the advent of targeted therapy and immunotherapy, the overall survival (OS) time of HCC patients has been improved to some extent (Ritchie et al., 2018; Wei et al., 2019). Nevertheless, seeking safe and effective targets or activating the cytotoxic function of effector immune cells in the tumor microenvironment (TME) is still a difficult issue for HCC patients during immunotherapy.

Epigenetic regulation opens a whole new level of molecular research for us, that is, post-transcriptional regulation independent of genomic DNA sequences (Dawson and Kouzarides, 2012; Hogg et al., 2020). Aberrant levels of epigenetic regulation mediate a variety of complex biological processes in tumor development and progression (Phillips et al., 2020; Hu et al., 2022). M6A methylation modification is the most prevalent RNA methylation modification and can mediate RNA cleavage and splicing at the post-transcriptional level affecting RNA stability (Dang et al., 2021; Jiang et al., 2021). RNA modified by m6A methylation could cause changes in expression levels after binding to specific binding proteins (Liu et al., 2020). Therefore, dysregulation of m6A methylation balance can lead to activation or inhibition of downstream functional target molecule expression, which in turn leads to the occurrence of malignant events such as tumor proliferation, distant metastasis, and treatment resistance (Han et al., 2019; Lin et al., 2020; Zhang et al., 2020). In m6A RNA methylation, mainly methyltransferase (termed “writer”), demethylase (termed “eraser”), and binding protein (termed “reader”), regulate the physiological or pathological processes of diseases (Lee et al., 2021). Previous studies have confirmed that m6A modifiers distinguish colorectal cancer (CRC) patients into four classes to characterize their tumor microenvironment and pharmacogenomic landscape (Chen et al., 2021b). Numerous studies have shown that methylation-related regulatory molecules are key markers of HCC and reflect differences in patient prognosis (Chen et al., 2018; Zhang et al., 2020; Zhou et al., 2020). Traditional bisulfite sequencing methods and RNA immunoprecipitation sequencing (RIP-seq) are not conducive to the dynamic detection of the patient’s condition and prognostic evaluation (Chen et al., 2018). Therefore, new feasible tools are urgently needed to achieve the effect of predicting prognosis and guiding clinical decision-making.

Herein, we collected 35 key m6A regulators and the corresponding transcriptome data and analyzed the pathological and immunological characteristics mediated in HCC patients *via* m6A manner. Subsequently, consensus clustering was performed based on the differences in m6A molecular expression patterns. Furthermore, we constructed an m6A risk score model and demonstrated the predictive effect of the model on prognosis and immune infiltration.

## Materials and methods

### Publicly available data acquisition and m6A regulator gene collection

Gene expression data and clinical information of the liver hepatocellular carcinoma cohort in UCSC Xena from The Cancer Genome Atlas (TCGA, https://portal.gdc.cancer.gov/) were collected and termed TCGA-LIHC cohort. Correspondingly, GSE14520, GSE27150, and GSE54236 from the Gene Expression Omnibus (GEO, http://www.ncbi.nlm.nih.gov/geo/) database and the liver hepatocellular carcinoma cohort from the International Cancer Genome Consortium (ICGC, https://dcc.icgc.org/) Data Portal were collected. The somatic mutation and copy number variation (CNV) data were downloaded from cBioPortal (http://cbioportal.org/). The RNA sequencing data were transformed into the transcripts per kilobase million (TPM) format. The R packages *affy* and *lumi* were run to normalize the batch effect. The inclusion criteria for the samples are as follows: 1) primary HCC; 2) gene expression profiles and related clinical information were available; 3) preoperative radiotherapy and chemotherapy were not performed. A total of 35 m6A methylation-related regulator genes (MRGs) were collected from previous studies (Zaccara et al., 2019; Chen et al., 2021b; Zhang et al., 2022). Genes were annotated and extracted using the Genome Reference Consortium Human Build 38 (GRCh38).

### Consensus clustering of 35 m6A regulator genes and different expression genes

Unsupervised cluster typing of 35 m6A regulators, namely, nine writers (VIRMA, METTL3, METL14, WTAP, RBM15, RBM15B, METL16, ZC3H13, and PCIF1), 23 readers (TRMT112, ZCCHC4, NUDT21, CPSF6, CBLL1, SETD2, HNRNPC, RBMX, HNRNPA2B1, IGF2BP1, IGF2BP2, IGF2BP3, YTHDC1, YTHDF1, YTHDF2, YTHDF3, YTHDC2, SRSF3, SRSF10, XRN1, FMR1, NXF1, and PRRC2A), and three erasers (FTO, ALKBH5, and ALKBH3) was explored using the *ConsensusClusterPlus* R package. Visualization results of cumulative distribution function (CDF) under different K-values (2–9) were analyzed. A random sample of 80% was selected for 500 repeated times of all genes. Subsequent unsupervised clustering of differentially expressed genes (DEGs) was described as mentioned previously. Furthermore, 30 criteria were performed in the *NbClust* R package to re-determine the optimal number of clusters.

### Somatic mutation and copy number alteration analysis

Somatic mutation and copy number alteration (CNA) information of HCC were downloaded from TCGA and cBioPortal (http://www.cbioportal.org) for cancer genomics. The *maftools* package was performed to summarize and annotate mutational information, including tumor mutation burden (TMB), single-nucleotide polymorphism (SNP), and insertion and deletion (INDEL) (Mayakonda et al., 2018). The *ComplexHeatmap* package converts the results into waterfall diagrams (Gu et al., 2016).

### Gene differential expression analysis

The TPM format of the TCGA-LIHC dataset was log2 logarithmic to enhance the comparability between different samples. Differentially expressed genes (DEGs) were obtained by *limma* package screening (Ritchie et al., 2015). Subsequently, genes with an absolute value of log2FoldChange > 1 and a false discovery rate (FDR) < 0.01 were rigorously defined as significant DEGs.

### Functional enrichment analysis

Based on the signaling pathways that belong to the Gene Ontology (GO) and Kyoto Encyclopedia of Genes and Genomes (KEGG) collected from MSigDB (http://www.gsea-msigdb.org/gsea/msigdb/index.jsp), we performed gene enrichment analysis for DEGs. The *clusterProfiler* package was used to calculate the normalized enrichment score (NES). When FDR was less than 0.01, the gene set was retained and considered to be significantly enriched. In GO analysis, 20 representative functional pathways in three categories (namely, biological process, cellular component, and molecular function) were selected for visualization, respectively.

### Assessment of immune cell infiltration

To demonstrate the global landscape of immune cell infiltration abundance, four algorithms were used to compare the infiltration features of immune cells, respectively. 1) Single-sample gene set enrichment analysis (ssGSEA) algorithm; the *GSVA* package was used to compare the abundance of 28 immune cells for each individual; 2) *xCell* package; signature extraction was performed among a total of 64 immune cells and stromal cells, including epithelial cells, hematopoietic progenitor cells, and extracellular matrix cells; 3) *MCP-counter* package; the abundance of eight immune cells and two stromal cells from normalized transcriptome data was calculated; 4) Tumor Immune Estimation Resource (TIMER, https://cistrome.shinyapps.io/timer/) database; the Spearman correlation between immune cell infiltration and gene expression was summarized. The *pheatmap* package was adopted to visualize the aforementioned results. Furthermore, we assessed the purity of tumor samples using the *ESTIMATE* package, which deduces the proportion of mesenchymal cells to immune cells in tumor tissues by analyzing gene expression profile characteristics.

### Generation of m6A gene signature

For the purpose of making the model easier to explore and enhancing clinical convertibility, the principal component analysis (PCA) was adapted to perform dimension reduction analysis on standardized gene expression data to preserve key features. Furthermore, the m6A score was calculated with the feature coefficients of principle components 1 and 2:

$$m6A\;score=\overset{n}{\underset{i=1}{\sum }}({PC1}_{i}+{PC2}_{i}).$$

Here, *i* refers to the m6A-related key genes and *n* is the total of m6A-related key genes.

The Sankey diagram was performed to represent the diverging situation between different clusters in proportion and finally reflected in different survival outcomes.

### Nearest template prediction validation

We used the *CMScaller* R package to calculate the nearest template prediction (NTP). The NTP algorithm provides a convenient method to make category predictions, using only a gene list and a test dataset to assess the predicted confidence calculated in each patient’s gene expression data. In order to intuitively demonstrate the prediction and grouping ability of the m6Ascore model, four validation queues were verified and the results were visualized: GSE14520, GSE27150, GSE54236, and ICGC cohorts. Among them, the intersection of module characteristic genes and DEGs was the feature genes of the NTP program.

### Immunotherapy prediction and drug sensitivity exploration

The tumor Immune Dysfunction and Exclusion (TIDE, http://tide.dfci.harvard.edu/) score and SubMap method were used to predict the potential efficacy of immune checkpoint inhibitor therapy (including anti-PD1 therapy and anti-CTLA-4 therapy) for individuals in each cluster. The *pRRophetic* package was applied to obtain the half-maximal inhibitory concentration (IC_50_) value of distinct drugs (Geeleher et al., 2014). The IC_50_ value can reveal the sensitivity of patients with different m6Ascores in HCC treatment to the ability of chemotherapy drugs to induce apoptosis.

### Statistical analysis

All analysis was performed in the environment of R (4.1.0 version) software. Kaplan–Meier and Cox regression in the *survival* package were used to analyze survival differences between groups and calculate the cumulative survival rate. The Pearson’s chi-squared test for categorical variables and t-test for continuous variables were used in this study. The spearman analysis was performed to calculate the correlation. The *circlize* package was used to transform results into visual circular shapes. The two-tailed method with *p* < 0.05 was deemed as statistically significant.

## Results

### Identification of 35 key m6A phenotype-associated regulators

**FIGURE 1:**
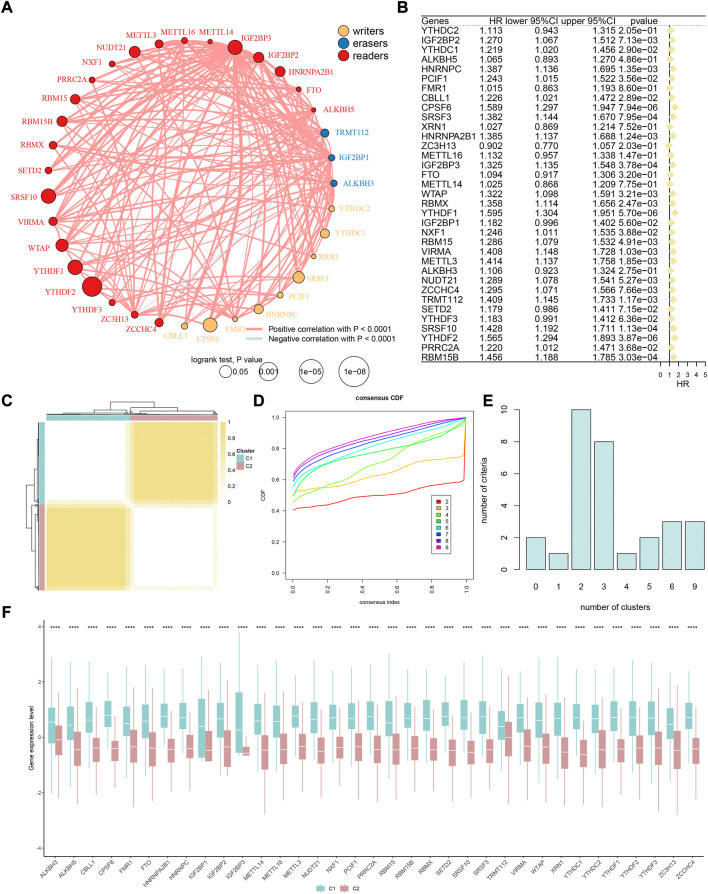
M6A methylation modification patterns in HCC. **(A)** Interaction between m6A regulators. Yellow dots represent writers, blue dots represent erasers, and red dots represent readers. Pink lines represent positive correlation between m6A regulators, and blue lines represent negative correlation between m6A regulators. The size of each circle represents the prognostic effect of each adjustment factor and is scaled by a *p*-value. **(B)** Univariate Cox regression analysis of 35 m6A regulators. **(C)** Consensus matrix when k = 2. **(D)** Consensus cumulative distribution function (CDF) of the consensus matrix for each k (indicated by colors). **(E)** Optimal cluster number by K-means clustering and program NbClust. **(F)** Difference in the expression level of 35 m6A regulators between two m6A clusters.

As the m6A methylation level is regulated by a series of enzymes and RNA-binding proteins *in vivo*, we recruited 35 m6A phenotype-related regulators based on existing studies (Zaccara et al., 2019; Chen H. et al., 2021; Zhang et al., 2022). The detailed names were listed in the *Methods* section. The flowchart of this study is shown in Supplementary Figure S1. First, the correlation and univariate analysis among 35 m6A regulators were performed in TCGA-HCC cohort (Figures 1A,B), which showed extensive crosstalk and widely prognostic implications. Most of these regulators are risk factors in liver cancer, such as IGF2BP2, YTHDC1, HNRNPC, PCIF1, CBLL1, CPSF6, SRSF3, HNRNPA2B1, IGF2BP3, WTAP, RBMX, YTHDF1, NXF1, RBM15, VIRMA, METTL3, NUDT21, ZCCHC4, TRMT112, SRSF10, YTHDF2, PRRC2A, and RBM15B (all *p* < 0.05, HR > 1). Meanwhile, SETD2 ranked first when comparing the somatic mutation frequency of 35 regulators (Supplementary Figure S2A). The CNA analysis revealed that VIRMA has the most copy number amplification, while YTHDF1 has the most copy number deletion (Supplementary Figure S2B). Additionally, the expression levels in 33 of the 35 regulators were significantly different between tumor and normal tissues (Supplementary Figure S2C). In brief, these regulators might exhibit strong bioregulatory potential.

### Construction and exploration of m6A-pattern cluster

**FIGURE 2:**
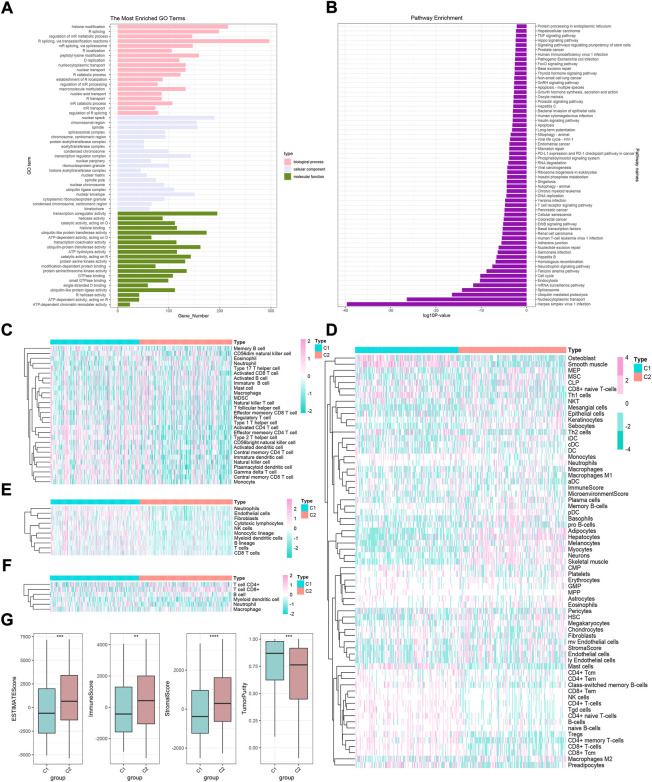
GO and KEGG analyses and immune cell infiltration characteristics in two m6A modification modes in HCC. **(A)** GO enrichment analysis. **(B)** KEGG enrichment analysis. **(C–F)** Heatmaps of immune cell infiltration in m6A modification patterns based on ssGSEA, xCell, MCP-counter, and TIMER algorithms, respectively. **(G)** Difference in ESTIMATE, immune, stromal, and tumor purity scores between two m6A modification modes based on ESTIMATE algorithm.

Previous studies have confirmed that m6A modification-related methylases, demethylases, and RNA-binding proteins play a fundamental role in the tumorigenesis and development of HCC (Zhou et al., 2020; Du et al., 2021; Kim et al., 2021; Li et al., 2021). Subsequently, TCGA-HCC cohort was divided into two clusters according to the expression of 35 m6A regulators: phenotypic-cluster 1 (C1, *n* = 180) and phenotypic-cluster 2 (C2, *n* = 188) (Figure 1C). Furthermore, the calculation of consensus CDF and program NbClust analyses suggested that K = 2 was the optimal cluster number (Figures 1D,E). Notably, patients in the two clusters showed considerable differences in gene expression (Figure 1F, all *p* < 0.0001). Gene set variation analysis (GSVA) was applied to explore the potential biological functions of m6A modification modes in two phenotypic-clusters (Figures 2A,B). The RNA splicing, histone modification, peptidyl-lysine modification, and nuclear envelope and transcription regulator activity in the GO term and the herpes simplex virus 1 infection, nucleocytoplasmic transport, ubiquitin-mediated proteolysis, spliceosome, and mRNA surveillance pathway in KEGG analysis were ranked ahead. To further demonstrate the infiltrating features of immune-related cells globally, four independent algorithms were used: ssGSEA, xCell, MCP-counter, and TIMER algorithm (Figures 2C–F). Also, the NES of cells included in the four algorithms also showed statistical differences between the two phenotypic-clusters (Supplementary Figures 3A–D). The fraction comparison of ESTIMATE score, immune score, stromal score, and tumor purity also showed statistical differences (Figure 2G, all *p* < 0.01), which manifested that the m6A modification pattern might shed light on the cellular level of the immune microenvironment and guide the immunotherapy in clinical practice of HCC patients.

### Screening of differentially expressed genes and establishment of gene clusters

**FIGURE 3:**
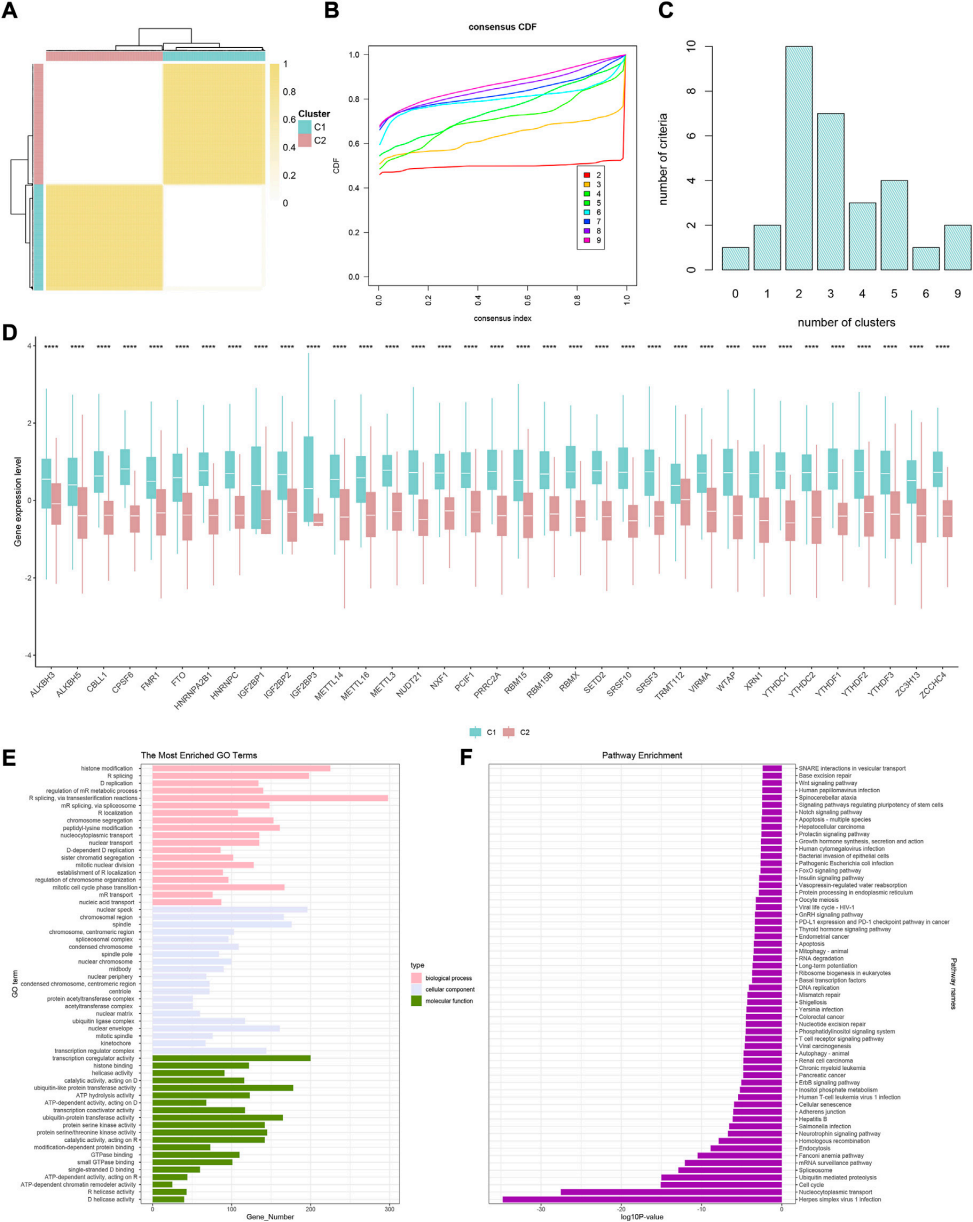
Two genomic subtypes of differentially expressed genes that can affect a cancer patient’s prognosis using unsupervised cluster analysis. **(A)** Consensus matrix when k = 2. **(B)** CDF graph of the consensus matrix for each k (indicated by colors). **(C)** Optimal cluster number by K-means clustering and program NbClust. **(D)** Difference in the expression level of 35 m6A regulators between two gene clusters (*p < 0.05, **p < 0.01, ***p < 0.001, ****p < 0.0001, and ns, not significant). **(E)** GO enrichment analysis. **(F)** KEGG enrichment analysis.

**FIGURE 4:**
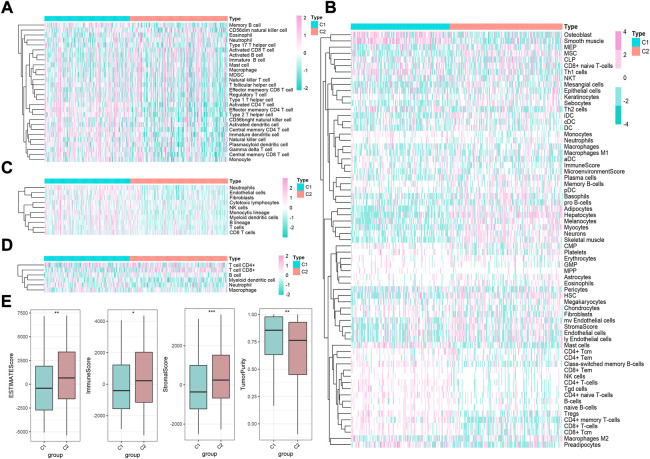
Immune landscape underlying two different m6A gene clusters in liver cancer. **(A–D)** Heatmaps of immune cell infiltration in m6A gene clusters based on ssGSEA, xCell, MCP-counter, and TIMER algorithms, respectively. **(E)** Difference of ESTIMATE, immune, stromal, and tumor purity scores between two m6A gene clusters based on ESTIMATE algorithm (*p < 0.05, **p < 0.01, ***p < 0.001, ****p < 0.0001, and ns, not significant).

Given the transcription imparity between m6A modifiers in distinct phenotypic-clusters, we explored the genetic characteristics and potential mechanisms regulated by m6A modes. First, 3500 DEGs were screened out between two m6A phenotypic-clusters. Furthermore, the remaining 2260 DEGs were filtered by univariate Cox analysis. Subsequently, two gene clusters (C1, *n* = 172; C2, *n* = 196) based on 2260 DEGs were obtained by unsupervised cluster analysis and verified (Figures 3A–C). Similarly, all 35 m6A regulators were significantly upregulated in gene-cluster 1 (Figure 3D). Moreover, the top pathways enriched by GO and KEGG analyses were roughly the same as the phenotypic-cluster results. Among them, the cell cycle pathway was worth mentioning and ranked third in KEGG (Figures 3E,F). The abundance of immune cell infiltration and the NES of related cells were evaluated for the two gene clusters, respectively (Figures 4A–D; Supplementary Figures 4A–D). The primary immune cells that dominate innate and/or acquired immunity in humanity, such as central memory CD8 T cells (ssGSEA method), effector memory CD4 T cells (ssGSEA method), cytotoxic lymphocytes (xCell method), NK cells (xCell method), macrophages (MCP-counter method), Th2 cells (TIMER method), and Tregs (TIMER method), were differently enriched in gene-clusters 1 and 2. The ESTIMATE score, immune score, stromal score, and tumor purity also showed statistical differences (Figure 4E, all *p* < 0.01).

### Generation of the m6Ascore model and detection of functional roles

**FIGURE 5:**
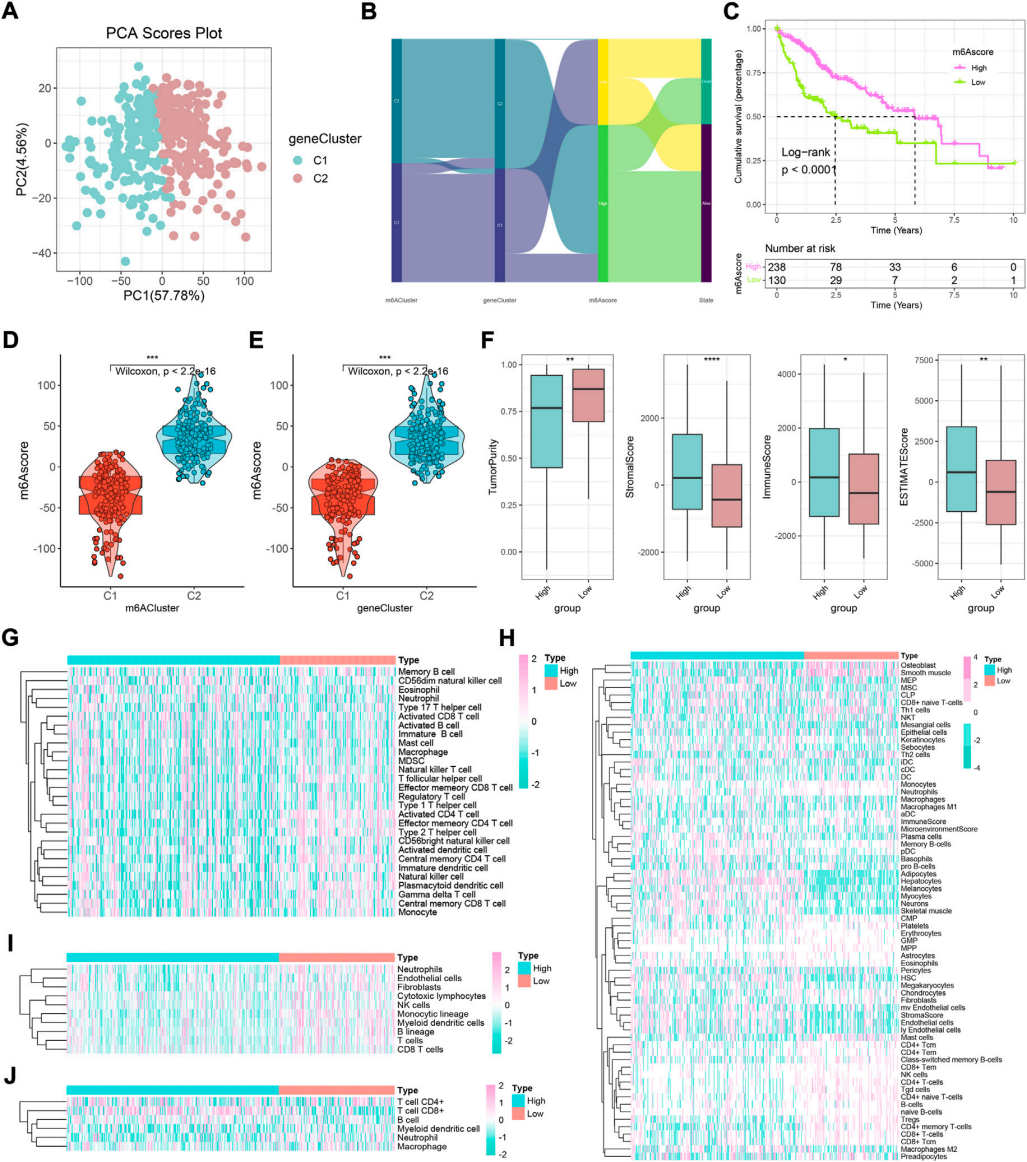
Construction of an m6A gene signature and exploration of its clinical significance in HCC. **(A)** Two gene clusters by principal component analysis. **(B)** Sankey diagram showing the changes in m6A clusters, gene clusters, m6Ascore, and survival status. **(C)** Survival outcomes of patients by m6Ascore. The survival outcomes of patients in the low-m6Ascore group are better. **(D)** Variation analysis of m6Ascore between m6A clusters. **(E)** Variation analysis of m6Ascore between gene clusters. **(F)** Difference in ESTIMATE, immune, stromal, and tumor purity scores between high- and low-m6Ascore groups based on ESTIMATE algorithm (*p < 0.05, **p < 0.01, ***p < 0.001, ****p < 0.0001, and ns, not significant). **(G–J)** Heatmaps of immune cell infiltration in high- and low-m6Ascore groups based on ssGSEA, xCell, MCP-counter, and TIMER algorithms, respectively.

In order to further refine this subject and achieve individualized prediction, we further explored the m6Ascore model based on gene clusters. The PCA showed well separation efficiency (Figure 5A). When comparing the diverging status of samples from different subtypes, it was found that the m6Ascore might have the best predictive power for prognosis, and low-m6Ascores possess adverse outcomes (Figures 5B,C, log-rank *p* < 0.0001). Interestingly, the high-m6Ascore subtype was positively correlated with m6A phenotypic-cluster 2 and gene-cluster 2 and was accompanied by low tumor purity, high stromal score, high ESTIMATE score, and high immune score (Figures 5D–F, all *p* < 0.05). Four calculations showed that low-m6Ascore patients generally possessed a higher abundance of local immune cell infiltrates, including the ssGSEA method (the activated CD4 T cell, central memory CD4 T cell, central memory CD8 T cell, effector memory CD4 T cell, immature dendritic cell, and type 2 T helper cell), MCP-counter method, TIMER method, and xCell method (CD4 + memory T cells, Th1 cells, and Tregs) (Figures 5G–J, Supplementary Figures 5A–D, all *p* < 0.05). Some immune cell infiltration was more superior in the high-risk group, while most of these made no sense and were not statistically significant (such as activated B cell, aDC, and cDC). Additionally, only several types of cells show different trends in xCell algorithm (such as DC and endothelial cells). However, there was no consistency among the four algorithms (ssGSEA, MCP-counter, TIMER, and xCell). Therefore, considering the analysis results of the four algorithms, it is reasonable to assume that the low-risk group may be more responsive to immunotherapy.

### Validation of nearest template prediction in the m6Ascore model and functional exploration

**FIGURE 6:**
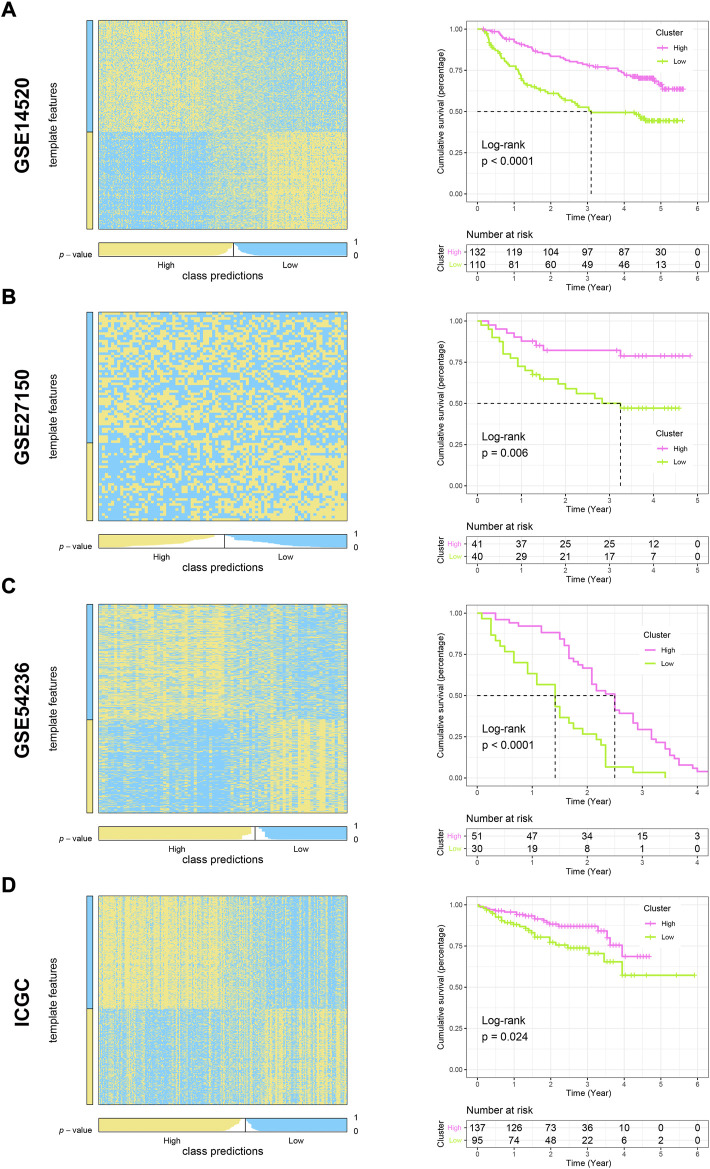
Validation of high- and low-m6Ascore grouping using the NTP algorithm and exploration of prognostic differences in other cohorts. **(A–D)** Heatmaps and K-M curves showing predicted patient distribution and prognostic differences for liver cancer in GSE14520, GSE27150, GSE54236, and ICGC cohorts, respectively.

**FIGURE 7:**
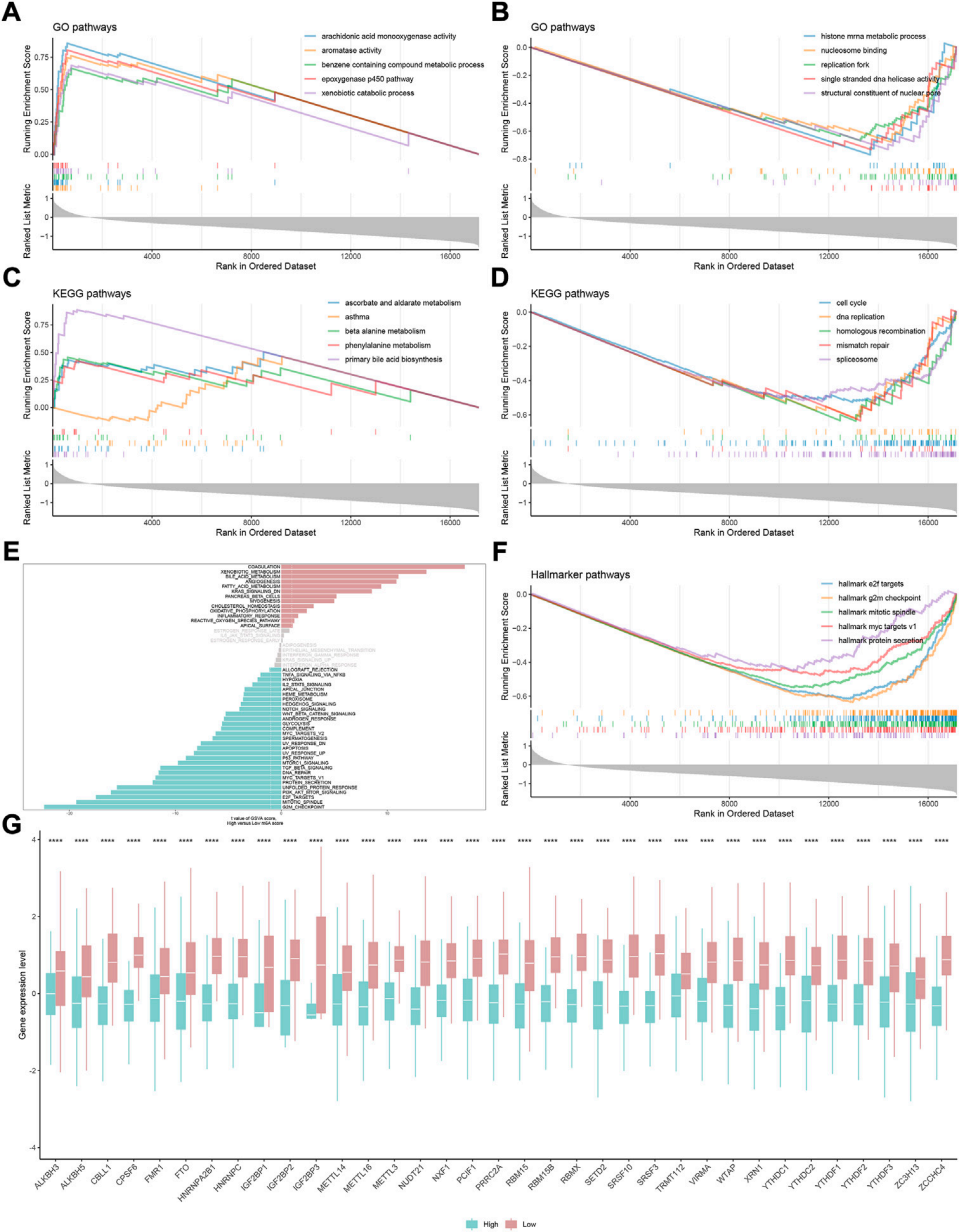
Gene set enrichment analysis underlying the m6A signature in HCC. **(A)** Most significantly enriched GO terms in the high-m6Ascore group. **(B)** Most significantly enriched GO terms in the low-m6Ascore group. **(C)** Most significantly enriched KEGG pathways in the high-m6Ascore group. **(D)** Most significantly enriched KEGG pathways in the low-m6Ascore group. **(E)** Gene set variation analysis underlying the m6A signature. **(F)** Most significantly enriched hallmark pathways in the low-m6Ascore group. **(G)** Difference in the expression level of 35 m6A regulators between high- and low-m6Ascore groups (*p < 0.05, **p < 0.01, ***p < 0.001, ****p < 0.0001, and ns, not significant).

To evaluate the reliability and robustness of the model, we performed nearest template prediction (NTP) and survival analysis in four queues recruited from different platforms: GSE14520, GSE27150, GSE54236, and ICGC cohort (Figures 6A–D). Patients in the low-m6Ascore subtype possess a poor prognosis in four validation datasets, respectively (all log-rank *p* < 0.05). The GSEA of the GO term found that the top five terms between high- and low-m6Ascore subtypes were distinct (Figures 7A,B). The metabolism-related terms of arachidonic acid monooxygenase activity, aromatase activity, benzene-containing compound metabolic process, epoxygenase p450 pathway, and xenobiotic catabolic process were visibly enriched in the high-m6Ascore subtype, while terms of histone mRNA metabolic process, nucleosome binding, replication fork, single-stranded DNA helicase activity, and structural constituent of nuclear pore were downregulated in the low-m6Ascore subtype. Similarly, the metabolism-related KEGG pathways were upregulated in high-score groups, such as ascorbate and aldarate metabolism, asthma, beta-alanine metabolism, phenylalanine metabolism, and primary bile acid biosynthesis pathways (Figure 7C). Remarkably, the gene regulation-correlated pathways were negatively enriched in low-score groups, such as cell cycle, DNA replication, homologous recombination, mismatch repair, and spliceosome pathways (Figure 7D). Furthermore, GSVA provided potential directions for m6A signatures to explore, such as G2M checkpoint, mitotic spindle, E2F targets, PI3K/AKT/MTOR signaling, coagulation, and corresponding hallmark pathways (Figures 7E,F).

### Comparison of transcriptome and genome level in distinct m6Ascore

Driving of the m6A phenotype is inextricably linked to genomics and transcriptomics. Undeniably, m6A regulators play a leading role in the occurrence of methylation modification events (Zaccara et al., 2019). Consequently, the gene expression level of all 35 regulators in each group of the m6Ascore model was compared. As expected, all 35 regulators showed absolute upregulation in low-m6Ascore patients (Figure 7G). Subsequently, the waterfall plot of 30 genes with the highest mutation frequency and the relationship between the model and mutation characteristics in HCC were performed. The mutation frequency of TP53 and CTNNB1 secured the top 2 ranks (Supplementary Figures 6A,B, both *p* < 0.05). The correlation analysis of mutation characteristics showed that the high-score group was associated with high mutation trends, including TMB, SNP, and INDEL (Supplementary Figures 6C–E).

### Prediction of immunotherapy responses and drug sensitivity prediction

**FIGURE 8:**
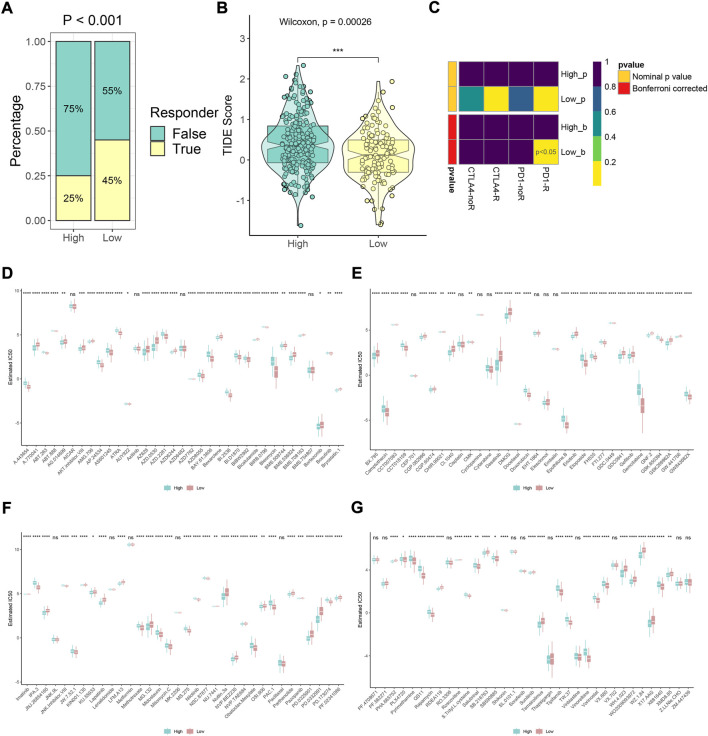
Immunotherapy response prediction and potential drug exploration. **(A,B)** Distribution of predicted immunotherapy responders and differences in TIDE scores based on TIDE algorithms in high- and low-m6Ascore groups. **(C)** Response prediction to immunotherapy (anti-PD-1 and anti-CTLA-4) between high- and low-m6Ascore groups according to the SubMap algorithm. **(D–G)** Difference in prediction of sensitivity of 138 drugs between high- and low-m6Ascore groups based on the pRRophetic algorithm (*p < 0.05, **p < 0.01, ***p < 0.001, ****p < 0.0001, and ns, not significant).

We further evaluated the prediction efficiency of the m6A model about immunotherapy effectiveness for HCC patients. Based on TIDE algorithms, the results showed the distribution of predicted immunotherapy responders and differences in TIDE scores in high- and low-m6Ascore groups (Figures 8A,B). The low-score subtype might be more sensitive to immunotherapy and possessed lower TIME score (*p* < 0.001). The SubMap method proved that low-m6Ascore patients receiving anti-PD-1 therapy may be effective, which contributed to the therapeutic strategies made for improving outcomes in HCC patients with low m6Ascore (Figure 8C, *p* < 0.05). Moreover, the IC_50_ values of 138 compounds were calculated using the *pRRophetic* package. The results revealed that high-m6Ascore individuals were sensitive to axitinib, bicalutamide, dasatinib, gefitinib, lapatinib, roscovitine, salubrinal, and sunitinib (Figures 8D–G, all *p* < 0.05), while ATRA, bleomycin, bosutinib, camptothecin, doxorubicin, etoposide, gemcitabine, nilotinib, rapamycin, shikonin, tipifarnib, and vorinostat might be sensitive on low-m6Ascore patients (Figures 8D–G, all *p* < 0.05).

## Discussion

Due to high heterogeneity, HCC has attracted extensive attention from clinicians and is also a difficult problem to be solved urgently in the field of oncology and immunology (Liu et al., 2021a; Liu et al., 2021b). Meanwhile, it cannot be ignored that the occurrence and progression of HCC often lead to a series of fatal complications, which will greatly increase the life safety threat of patients (Pinter et al., 2016; Marasco et al., 2019). Consequently, the complex pathogenesis and the obscure background of tumor local immune cell infiltration make it difficult to evaluate and treat HCC. Currently, more robust and comprehensive prognostic assessment and efficacy prediction systems are needed to help manage HCC patients.

Proverbially, m6A epigenetic modification has influenced the way and future directions of oncology research (Topper et al., 2020; Guo et al., 2022). As the methylated regulatory network mediated via m6A is closely linked to a variety of star pathways, such as the Hippo pathway, TGF-β pathway, and WNT pathway, m6A-related regulators have been endowed with increasing biological functions (Han et al., 2020; Feng et al., 2021; Qiao et al., 2021). For m6A modification, a reversible and dynamic process, perhaps the molecular network that dominates its global nature could clearly dissect its potential biological significance. METTL3-mediated m6A modification can promote the progression and metastasis of CRC and reduce the prognosis of patients (Li et al., 2019; Chen et al., 2021a). The LINC00460/DHX9/IGF2BP2 complex promotes CRC proliferation and metastasis by m6A modification, which could serve as a promising predictive biomarker for the diagnosis and prognosis (Hou et al., 2021). ALKBH5, a demethylase, impairs the malignancy of HCC *via* m6A manner and provides therapeutic targets of treatment (Chen et al., 2020). Attractively, Shen et al. (2021) identified that YTHDF1-related m6A modification-dependent ferroptosis is a potential target for the treatment of liver fibrosis. Another study demonstrated that WTAP was upregulated in HCC, which indicated the poor outcomes (Chen et al., 2019). Overall, these m6A regulators are closely associated with the prognosis of patients with solid tumors such as HCC. However, most studies have focused on individual methylation-related enzymes and/or RNA-binding proteins, and little is known about global m6A methylation regulatory networks and their applications. Likewise, the potential roles of m6A patterns in HCC patients are pendent.

In this study, we describe a methylation regulatory network by recruiting 35 m6A regulators: nine writers, 23 readers, and three erasers that have been reported in the previous literature works. For instance, METTL3 and METTL14, as methylating enzymes, regulate a variety of malignant events, including tumor proliferation, metastasis, stemness maintenance, and chemotherapy resistance (Li et al., 2019; Wang et al., 2020b; Xu et al., 2021; Zhang et al., 2021). The demethylase FTO, an “eraser” of m6A methylation, exhibits dual effects (tumorigenesis and tumor inhibition) in disparate solid tumors (Tao et al., 2021; Huang et al., 2022). In addition, FTO inhibitors and their homologues have shown certain therapeutic effects in clinical trials (Huang et al., 2015). Furthermore, univariate analysis showed that individual methylation levels were associated with different outcomes. Therefore, the degree of m6A modification was preliminarily classified according to the expression level of 35 regulators. The two clusters of the m6A pattern showed well differentiation at both genomic mutation and transcriptome levels. Enrichment analysis found that the genomic and metabolic regulation differences between phenotypic-clusters 1 and 2 are primarily reflected in the RNA splicing, histone modification, transcription coregulator activity terms, herpes simplex virus 1 infection, and nucleocytoplasmic transport pathways. Abundance analysis of four immune infiltrate statuses further explored the potential differences in molecular mechanisms and cell composition under different m6A modes.

Furthermore, we screened potential functional gene targets for different m6A phenotypic-clusters. A total of 2260 DEGs with survival significance from two phenotypic-clusters were obtained. The following clusters of differential genes showed consistent results with phenotypic-clusters in terms of expression of 35 regulators. Enrichment analysis and immune cell infiltration prediction also confirmed differences in metabolic and infiltration abundance between gene-clusters 1 and 2. ESTIMATE score was used to infer tumor cell composition and normal cell (mainly stromal cells and immune cells) infiltration to predict differences in tumor microenvironments. The result found that gene-cluster 1 has low immune and stromal cell infiltration and high tumor purity, which might be associated with tumor proliferation and drug resistance. To increase the usefulness of individual patient assessment, we reduced the dimension of data and constructed a m6Ascore model. Also, the model was closely associated with patient prognosis, m6A phenotypic-clusters, and gene clusters. The stability of the model for prognostic assessment was also verified by four external public datasets, namely, GSE14520, GSE27150, GSE54236, and ICGC cohort. Additionally, GO and KEGG analyses showed that metabolization-related pathways were significantly upregulated in the high-score group. The E2F targets, G2M checkpoint, mitotic spindle, MYC targets, and protein secretion hallmark pathways were most significantly enriched in the low-m6Ascore group.

For decades, immunotherapy has revolutionized cancer treatment. However, not all cancer patients can achieve satisfactory results after immunotherapy (Wang et al., 2020a; Liu et al., 2022). As a whole, immunotherapy strategies primarily based on immune checkpoint blockers still need to be investigated. How to screen out suitable immunotherapy for patients and select appropriate drugs for patients has become a prevailing challenge for clinicians. Herein, we explored the predictive power of the m6Ascore model for the treatment of HCC patients. It was found that patients in the low-m6Ascore group may have a greater chance of responding to immunotherapy, such as anti-PD-L1 therapy, than those in the high-m6Ascore group. Meanwhile, the IC_50_ values of individuals were calculated and showed that the IC_50_ values of some drugs, including axitinib, bicalutamide, dasatinib, and gefitinib, were smaller in the high-m6Ascore group, which indicated that these patients may be more sensitive, while some drugs, such as bosutinib, camptothecin, etoposide, nilotinib, and tipifarnib, might provide therapeutic effects on low-m6Ascore patients. In fact, some drugs, such as sunitinib and axitinib, have been proved to have good application prospects in HCC. Synergizing sunitinib and radiofrequency ablation to treat HCC triggered an antitumor immune response (Qi et al., 2020). Moreover, according to a clinical study, avelumab in combination with axitinib can be used as a first-line treatment in patients with advanced HCC (Kudo et al., 2021). Generally, the drug prediction results provide a broad clinical prospect for future treatment options.

## Conclusion

The m6A methylation-related regulators were adopted to progressively construct the phenotypic-cluster, gene cluster and m6Ascore model, and the differences between genomics and transcriptomics as well as the potential functional pathways and mechanisms were evaluated, respectively. Furthermore, immunotherapy based on epigenetic modification in clinical patients has been predicted, and some potential therapeutic compounds have been developed. In fact, there are some limitations to this study. First, all the data analyzed in this study are based on the public cohort, and more sample information and clinical in-house cohorts need to be further mined. Second, some related basic experiments would be more conducive to the verification of results *in vivo* and/or *in vitro*. Collectively, this study established a powerful m6Ascore model and proposed a stratified precision strategy, which contributed to achieving the classification management and enhancing prognosis for HCC patients.

## Data Availability

The datasets presented in this study can be found in online repositories. The names of the repository/repositories and accession number(s) can be found in the article/Supplementary Material.

## Supplementary material

SUPPLEMENTARY FIGURE S1 Workflow of our study.

SUPPLEMENTARY FIGURE S2 Landscape of genetic and expression variation of 35 m6A regulators in HCC.

SUPPLEMENTARY FIGURE S3 Normalized enrichment score (NES) of immune-infiltrating cells in two m6A modification patterns based on ssGSEA, MCP-counter, TIMER, and xCell, respectively.

SUPPLEMENTARY FIGURE S4 Expression of immune-infiltrating cells in two m6A gene clusters based on ssGSEA, MCP-counter, TIMER, and xCell, respectively.

SUPPLEMENTARY FIGURE S5 Expression of immune-infiltrating cells in high- and low-m6Ascore groups based on ssGSEA, MCP-counter, TIMER, and xCell, respectively.

SUPPLEMENTARY FIGURE S6 Mutation landscape underlying m6A signature.

## Abbreviations

HCC, hepatocellular carcinoma; TMB, tumor mutation burden; SNP, single-nucleotide polymorphism; CNA, copy number alteration; INDEL, insertion and deletion; OS, overall survival; m6A, N6-methyladenosine; MRGs, m6A methylation-related regulator genes; DEGs, differentially expressed genes.
