# Supplementary figures and images for: M6A regulator-mediated immune infiltration and methylation modification in hepatocellular carcinoma microenvironment and immunotherapy
Source: Front Pharmacol. 2022 Nov 10;13:1052177. doi: 10.3389/fphar.2022.1052177 (PMC9685318; doi:10.3389/fphar.2022.1052177)

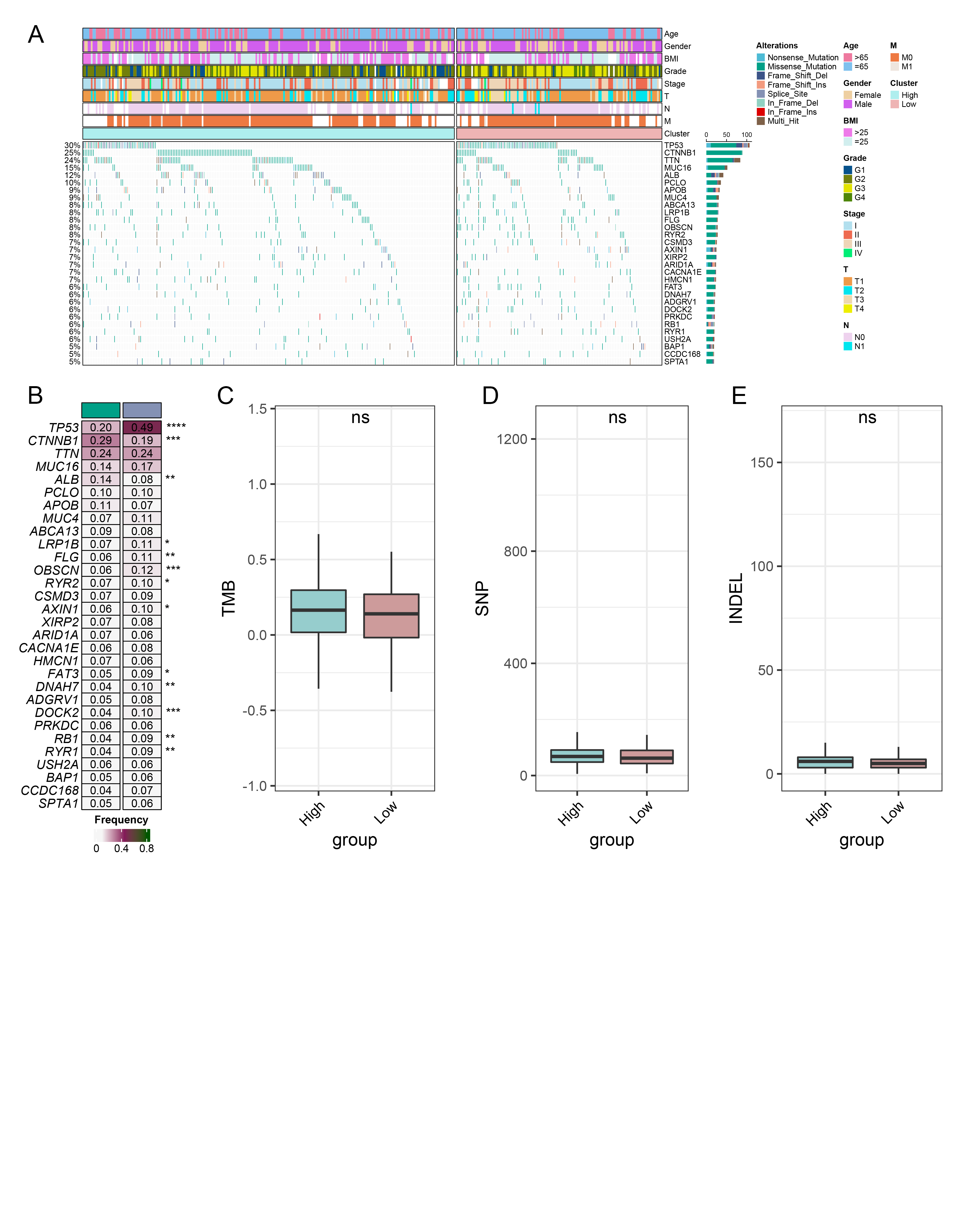

Supplement: Supplementary file 1 [file Image6.TIF]

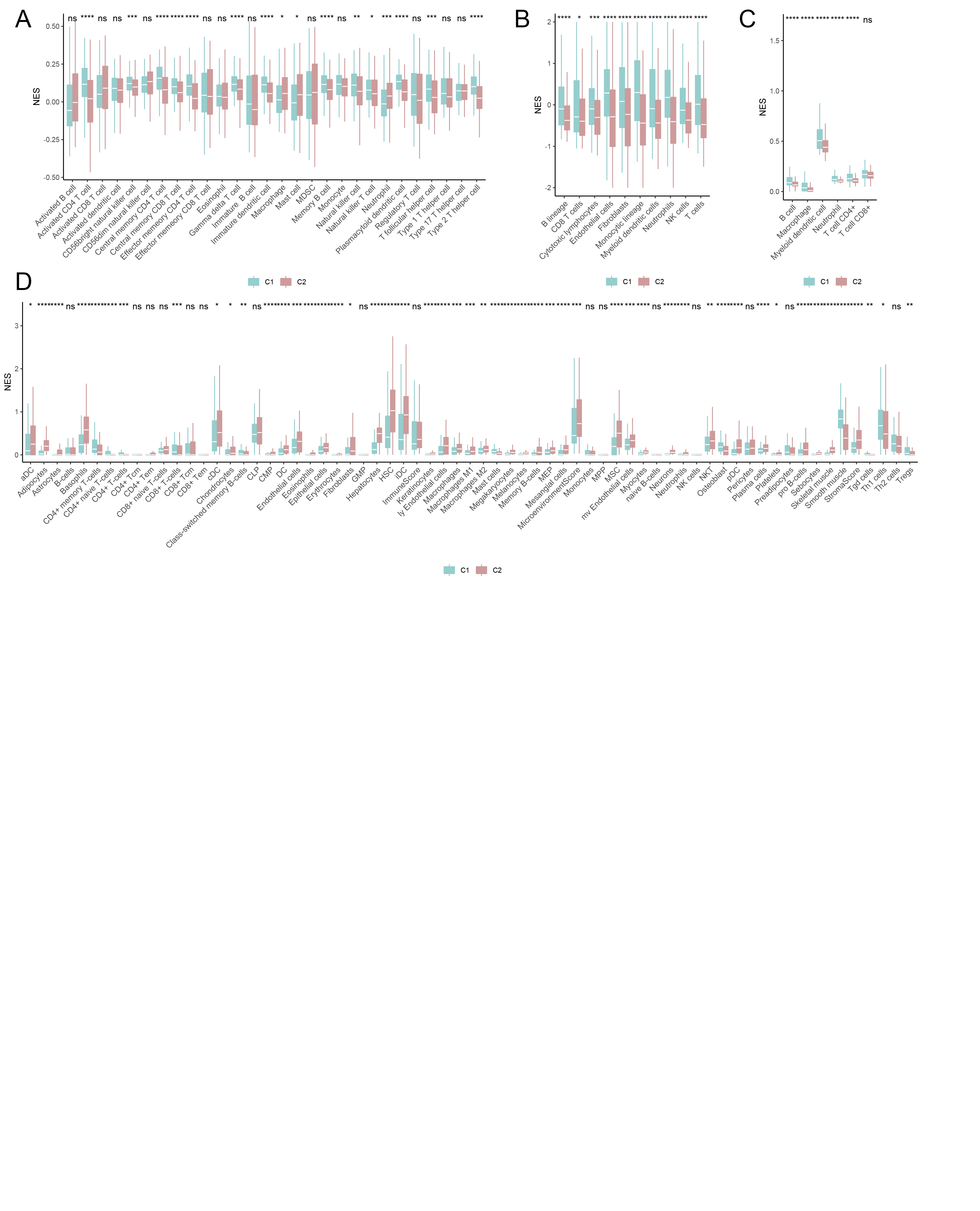

Supplement: Supplementary file 2 [file Image3.TIF]

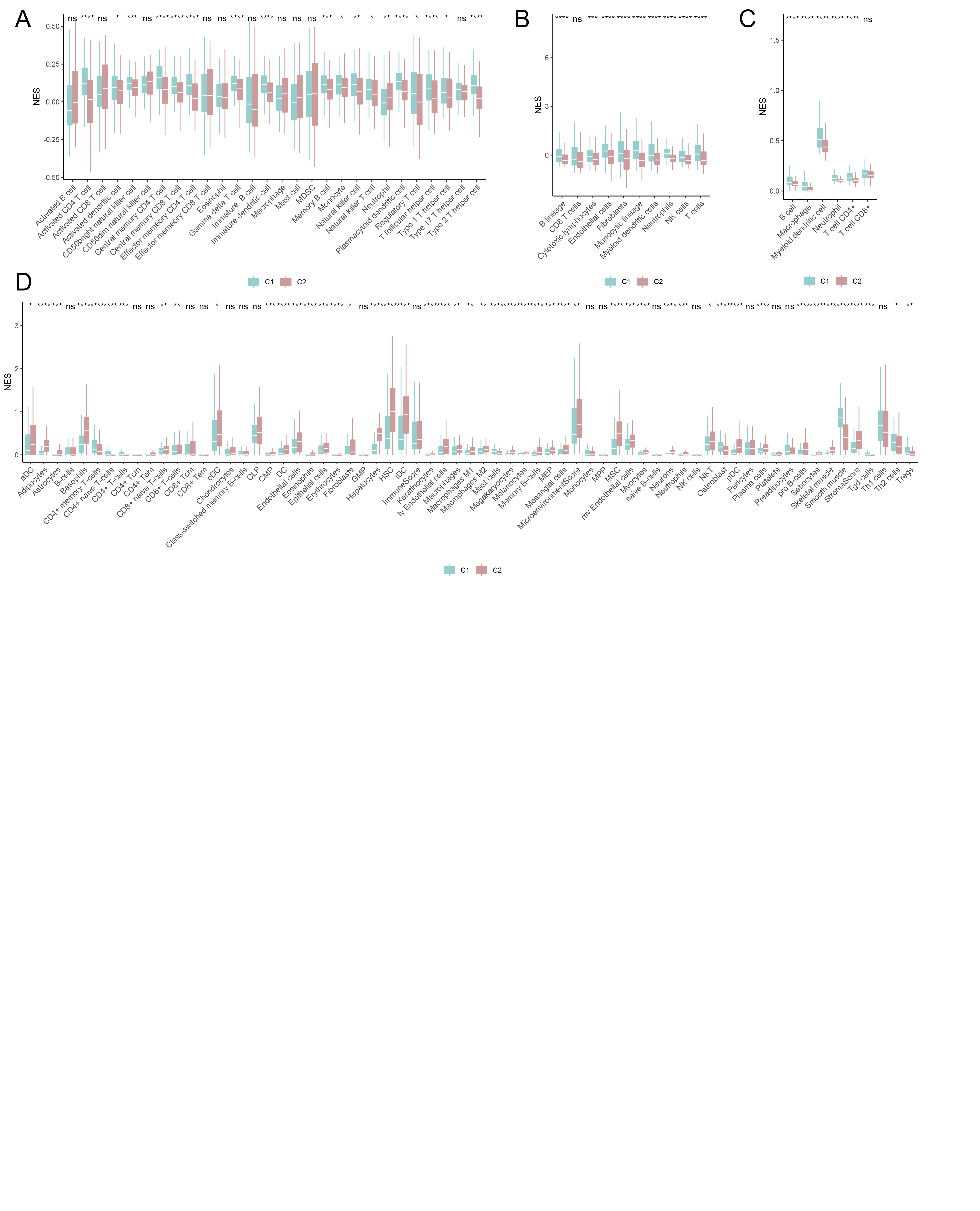

Supplement: Supplementary file 3 [file Image4.TIF]

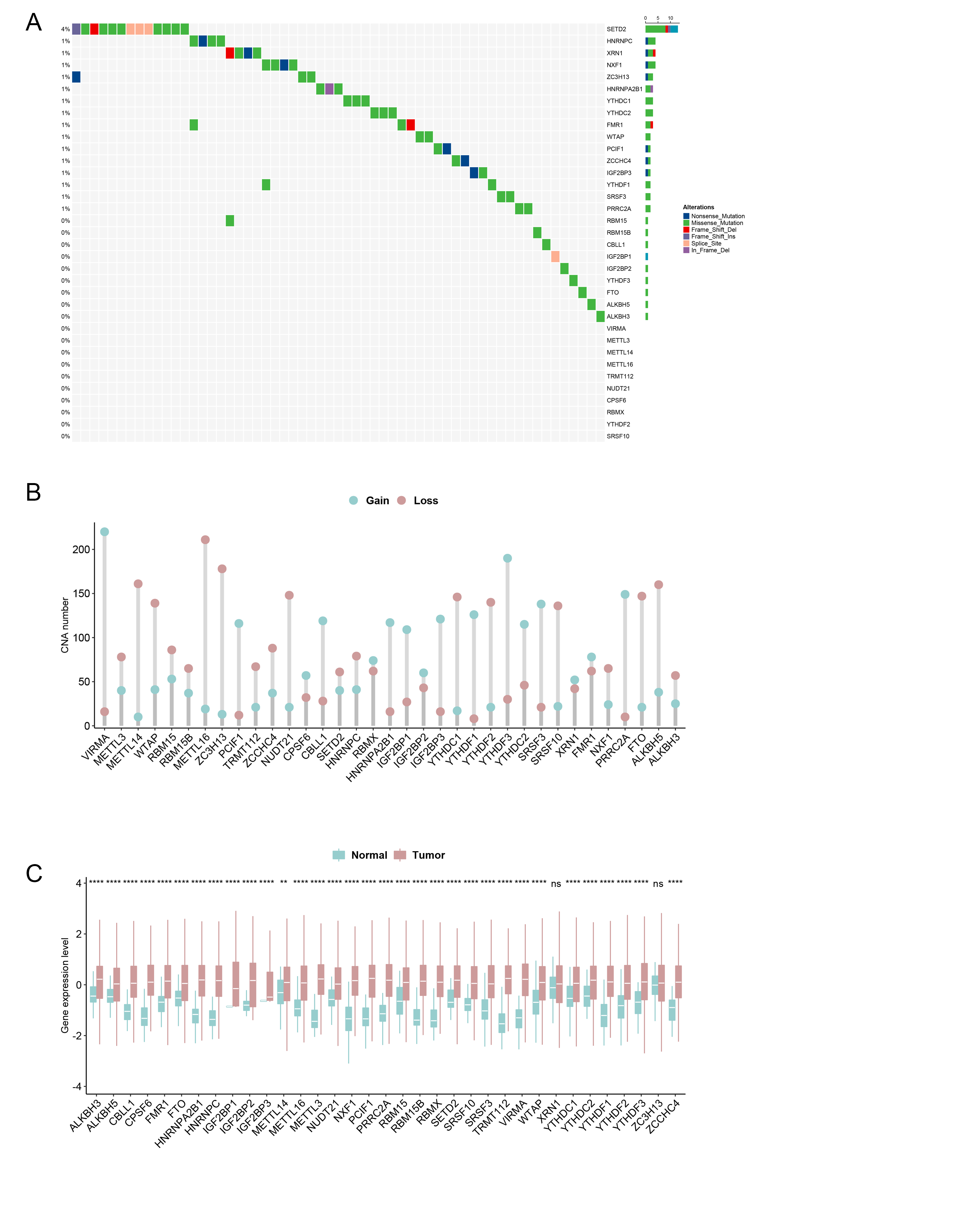

Supplement: Supplementary file 4 [file Image2.TIF]

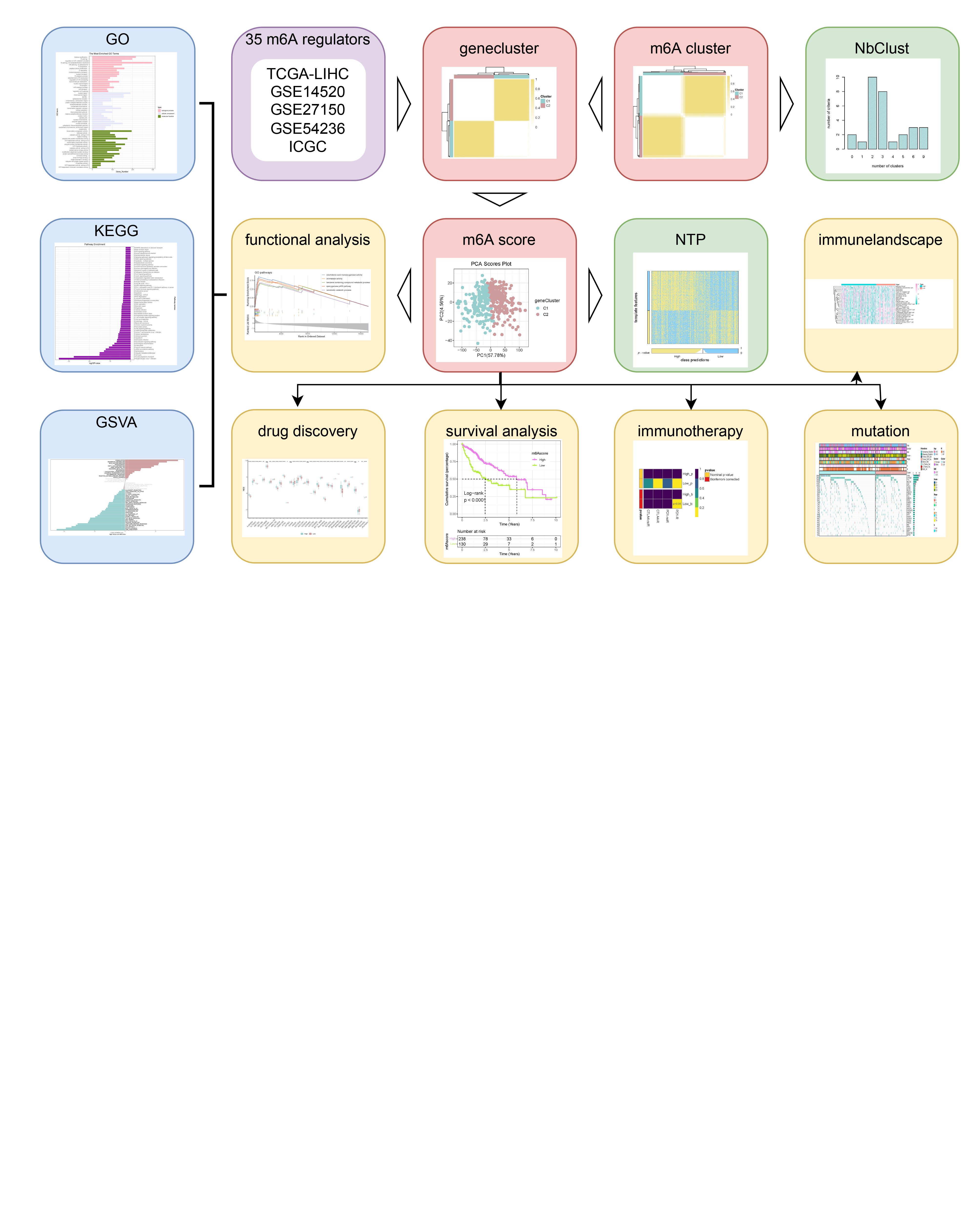

Supplement: Supplementary file 5 [file Image1.TIF]

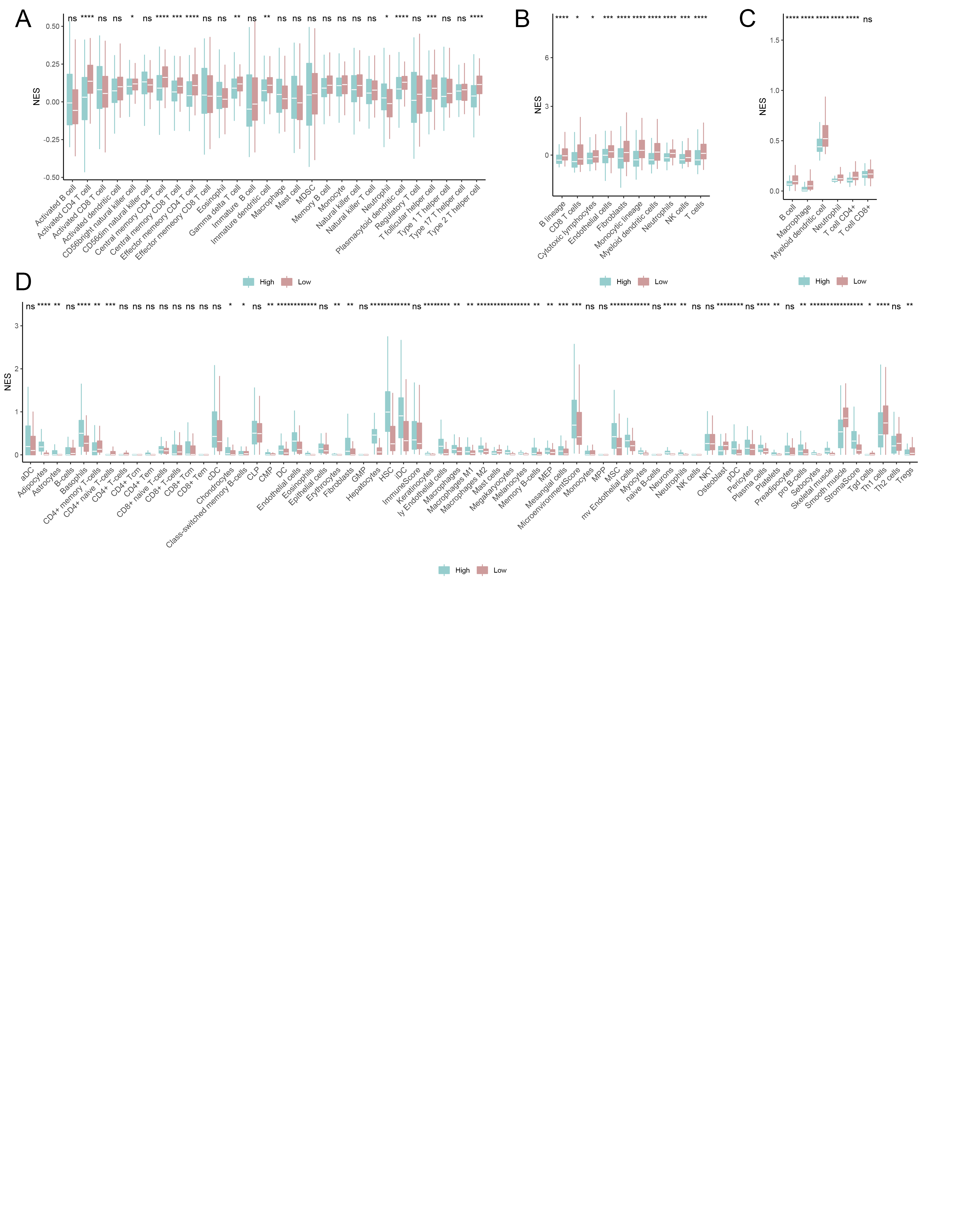

Supplement: Supplementary file 6 [file Image5.TIF]
